# A Broadband Photodetector Based on PbS Quantum Dots and Graphene with High Responsivity and Detectivity

**DOI:** 10.3390/nano13131996

**Published:** 2023-07-02

**Authors:** Mutan Luo, Run Chen, Zhaowei Zhu, Chuantong Cheng, Xin Ning, Beiju Huang

**Affiliations:** 1 State Key Laboratory on Integrated Optoelectronics, Institute of Semiconductors, Chinese Academy of Sciences, Beijing 100083, Chinaningxin@semi.ac.cn (X.N.);; 2 Brain Machine Fusion Intelligence Institute, Suzhou 215133, China; 3 College of Science, China Agricultural University, Beijing 100083, China; 4 College of Materials Science and Opto-Electronic Technology, University of Chinese Academy of Sciences, Beijing 100049, China

**Keywords:** PbS quantum dots, graphene, indium tin oxide, photodetector, high responsivity and detectivity

## Abstract

A high-efficiency photodetector consisting of colloidal PbS quantum dots (QDs) and single-layer graphene was prepared in this research. In the early stage, PbS QDs were synthesized and characterized, and the results showed that the product conformed with the characteristics of high-quality PbS QDs. Afterwards, the photodetector was derived through steps, including the photolithography and etching of indium tin oxide (ITO) electrodes and the graphene active region, as well as the spin coating and ligand substitution of the PbS QDs. After application testing, the photodetector, which was prepared in this research, exhibited outstanding properties. Under visible and near-infrared light, the highest responsivities were up to 202 A/W and 183 mA/W, respectively, and the highest detectivities were up to 2.24 × 10^11^ Jones and 2.47 × 10^8^ Jones, respectively, with light densities of 0.56 mW/cm^2^ and 1.22 W/cm^2^, respectively. In addition to these results, the response of the device and the rise and fall times for the on/off illumination cycles showed its superior performance, and the fastest response times were approximately 0.03 s and 1.0 s for the rise and fall times, respectively. All the results illustrated that the photodetector based on PbS and graphene, which was prepared in this research, possesses the potential to be applied in reality.

## 1. Introduction

With the rapid development of the contemporary information industry, photoelectronic and microelectronic technology have become intertwined and supplement each other, which means that photoelectronic detection systems occupy a very important position in the modern information industry. Photodetectors can convert different types of optical signals into electrical signals for output, transmission, imaging, and control feedback combined with signal processing. Therefore, photodetectors are widely used in various fields. Visible photodetectors are mainly used in industrial automatic control, photometric measurement, and other field applications. However, under specific work conditions, infrared detection technology can work better due to its excellent environmental adaptability; hence, it is widely used in the military, national defense, and civil fields [1,2,3,4]. In conclusion, the increasing application demand has accelerated the research and development of photoelectric detection.

To date, there are many materials that have been used to manufacture photodetectors, including traditional HgCdTe photodetectors, quantum wall infrared photodetectors, and type-II strained layer superlattice, and quantum dot (QD) infrared photodetectors [5,6,7,8]. The low-dimensional nanocrystallization of these materials can effectively improve the response and detection rate of the devices, marking a new trend in the development of optoelectronic materials. In addition, the device structure of the photodetector is also very important for its function; there are studies showing that the infrared focal plane array device with a flexible and adjustable response wave band is the core of the latest generation detector [9,10]. Therefore, the new nanosemiconductor optoelectronic materials, which are compatible with the existing, mature integrated device technologies, are the development direction for the photodetector [11]. Although the traditional detection materials possess good photoelectric properties in detector application, their high cost and complex manufacturing technology are obstacles to their development. In terms of detection sensitivity, photodetectors based on QD semiconductors can compete with inorganic semiconductors, such as Si and Ge and traditional photodetectors, which depend on high-temperature vacuum epitaxy. QDs are low-dimensional semiconductor materials. Their shape is generally spherical or quasispherical, and their diameter is often between 2 and 100 nm. QDs are usually composed of IV, II–VI, IV–VI or III–V elements which possess many characteristics that macro-materials do not, such as the quantum confinement effect, surface effect, and nano-size effect [12,13,14,15]. They show many physical and chemical properties that are different from those of macro-bulk materials. For instance, they are sensitive to incident light, synthesized in solution, and have no requirements for lattice matching [16]. In particular, when applying a certain electric field or light pressure to this nano-semiconductor material, they will emit light of a specific frequency, and the frequency of the light will change with the change in the size of the QDs. Therefore, the emission and absorption spectrum range of QDs can be controlled by adjusting their size [17,18,19]. Due to the above characteristics, QDs are becoming more widely used [20,21,22], but their application scope still has room for further expansion [11]. The common solution strategy is to expand the spectral response range by introducing inorganic substances, such as PbS. The peak response wavelength of a PbS QD photodetector can be adjusted in the range of 600 nm–3000 nm, making it an ideal short-wavelength photodetector [23].

At present, there are many physical and chemical methods for preparing PbS QDs. Physical methods, such as epitaxial growth, lithography, and etching, are restricted due to their ultra-fine machining process and high cost. Compared with physical methods, the chemical preparation method has the advantages of a simple process, controllable grain size, and being low cost, on account of which this method is used widely. The QDs produced from chemical preparation methods possess good dispersion and controllability in terms of size and morphology [24]. In addition, the surface of the product is coated with long-chain ligands, and the role of long-chain ligands is to control the nucleation and growth kinetics of QDs. Moreover, the ligand can maintain the stability and regulate the physicochemical properties of the QD product. These features provide the product with a good solution processability and ability to form a film at room temperature [25]. However, the long-chain ligand is insulated, and this results in the low carrier mobility of the corresponding device; thus, it is not suitable for application in photoelectric devices. As a result, the use of an uninsulated material or short-chain ligand as a substitute for the long-chain ligand is very important for the application of QDs in the field of photoelectric devices [26,27].

The response of a QD to incident light is firstly reflected in the change in its conductivity, which is the basis for photodetectors’ preparation. The electrons in the photogenerated electron–hole pair can be captured using the photosensitive center in QDs, and the captured electrons can generate additional signals, which leads to a significant increase in photoconductivity. However, the performance of a simple QD detector is not sufficient due to the low carrier mobility of QDs; therefore, other materials must be introduced to increase the electrical performance of the device. Graphene is a typical two-dimensional layered material, and it has great potential in the manufacture of photodetectors because of its high carrier mobility. However, graphene has the disadvantages of weak light absorption, fast carrier recombination, and an insufficient gain mechanism, which makes the performance of the devices based on graphene lower. For the above reasons, QDs and graphene can be combined to enhance the performance of photodetector, i.e., because their different work functions and carrier concentrations can achieve photoinduced charge transfer. Furthermore, at present, most photodetectors are still based on silicon. However, the energy consumption and heat generation of devices have become the bottleneck of the development and integration of traditional silicon-based devices. Meanwhile, the preparation of the source and drain electrodes of the traditional silicon-based photodetectors is usually by magnetron sputtering or the vacuum evaporation of a noble metal, which increases the preparation cost. Hence, other alternative schemes are explored. Indium tin oxide (ITO) is a kind of transparent brown film or yellowish gray block and is widely used to manufacture organic light-emitting diodes and photodetectors because of its high electronic transmission capacity, good light transmittance, and lower cost. Considering all the above factors, in this research ITO and graphene were used as the electrode and charge transport layer, respectively, and PbS QDs were used as photosensitive layer to prepare the photodetector, which could realize the efficient detection of both visible and near-infrared light.

In this research, 1-octadecene (ODE) acted as both surfactant and solvent, lead oxide (PbO) and bis (trimethylsilyl) sulfide ((TMS)_2_S) were used as the lead source and sulfur source, respectively, and oleic acid (OA) was used as the long-chain ligand to synthesize PbS QDs. Because the PbS QD product possess good film-forming characteristic, the long-chain ligand of the original product was substituted for ethylene dithiol (EDT) on the surface of the product in film state. The differences between the products before and after substituting were examined by various characterization methods. Besides PbS QDs, graphene was also introduced into the structure of the device to enlarge the photoelectric response. The photodetector was prepared on ITO quartz substrate by photolithography, etch, spin coating, and ligand substitution. The properties of the device were detected by distinct testing approaches.

## 2. Materials and Methods

**Figure 1:**
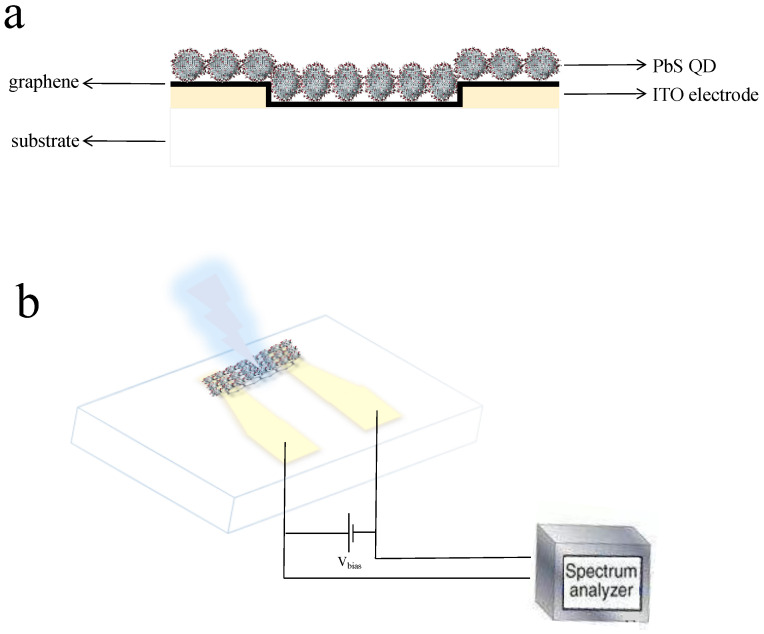
The structure (**a**) and mechanism (**b**) of the photodetector.

1.2 g PbO (Shanghai Aladdin chemical reagent Co., Ltd., Shanghai, China), 3 mL OA (Tianjin Damao chemical reagent Co., Ltd., Tianjin, China), and 20 mL ODE (Tianjin Damao chemical reagent Co., Ltd., Tianjin, China) were stirred at 90 °C with a vacuum background until the system formed a light yellow solution, which was used as the Pb precursor. 0.3 mL (TMS)_2_S (Shanghai Aladdin chemical reagent Co., Ltd., Shanghai, China) and 10 mL ODE were also stirred at room temperature with a vacuum background, and the solution was used as the S precursor. Then, the S precursor was injected into the Pb precursor, and before this operation, the reaction temperature had been raised to 160 °C. After the injection, the reaction temperature was set to room temperature. All the above steps operated under an O_2_-free and N_2_-filled environment. When the system temperature reached to room temperature, the PbS QDs product was washed with methanol several times and redispersed in hexane. The original ITO glass (Beijing Baichengtianyi technology Co., Ltd., Beijing, China) was graphically processed by photolithography and HCl (Tianjin Damao chemical reagent Co., Ltd., Tianjin, China) etching to prepare the ITO electrodes. Firstly, the ITO glass was cleaned using ultrasonic baths with organic solvents and deionized water, respectively, then the cleaned glass was processed by photolithography through a mask. Later, the glass was etched by dilute HCl to present the electrode. After the ITO electrodes were prepared, graphene (prepared from our lab) was transferred onto the substrate and graphically processed by photolithography through a mask and etched by oxygen plasma to form the active region. After all the above steps were completed, the PbS QDs solution was spin coated on the substrate to form a film, and a ligand substitution was performed by soaking the film in EDT solution (2 vol% in acetonitrile, Tianjin Damao chemical reagent Co., Ltd., Tianjin, China) for 30 s, followed by three rinse–spin cycles with acetonitrile (Tianjin Damao chemical reagent Co., Ltd., Tianjin, China). The PbS QDs were characterized using transmission electron microscopy (TEM) (FEI, CA, USA), X-ray diffraction (XRD) (Bruker, Karlsruhe, German), and NIR absorption spectrum (Nicolet, CA, USA). The device was tested by photoelectric test platform in our lab. Figure 1 showed the structure and the mechanism of the photodetector.

## 3. Results

### 3.1. The Synthesis and Characterization of PbS QDs

**Figure 2:**
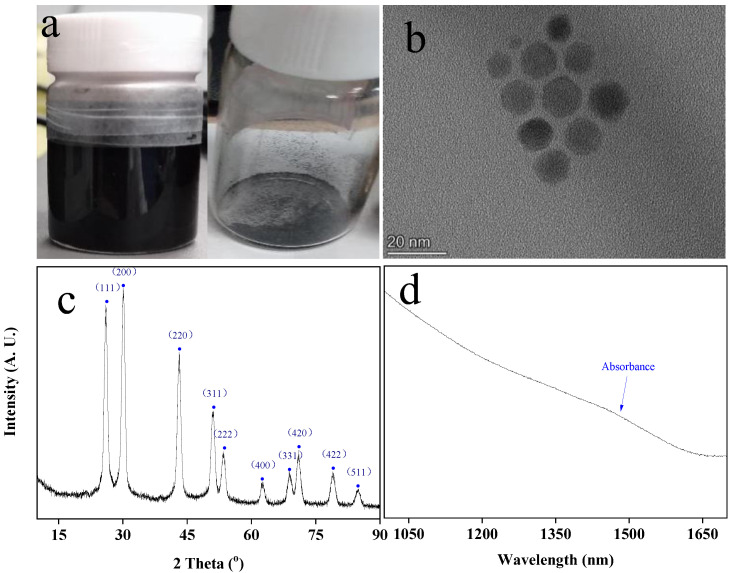
The characterizations of the PbS QDs synthesized in this research: (**a**) the appearance; (**b**) the micro morphology (accelerating voltage 200 KV; point resolution 0.25 nm; magnification 500 K); (**c**) the crystallinity (40 KV, 40 mA, and Cu kα λ = 1.5406 Å); (**d**) the absorption in the near-infrared range (wavelength range 400–2800 nm; scanning speed 600 nm/min).

The morphology and structure of PbS QDs are very important for the preparation of the photodetector. For this reason, the morphology and crystallization of the PbS QD product were characterized. The apparent morphology of the product is shown in Figure 2a. The dry sample is a black powder, and it changes to a black solution state in hexene. This shows that PbS QDs have good solubility in the solvent. The micromorphology of the QDs was characterized using TEM. Before the characterization, the QD sample was dispersed in the solvent and subjected to ultrasonic treatment to form a suspension. Afterwards, the sample suspension was dropped on a copper mesh with a supporting film. After the sample was dried, it was characterized using TEM. The shape of the product was observed to be spherical and appeared symmetrical. The edge of the shape contour was smooth. Additionally, the size distribution of the product was uniform, which indicates that the prepared PbS QDs possesses excellent monodispersity (Figure 2b). This is conducive to the stability of film formation and the consistency of the band gap. More importantly, the material with uniform size is beneficial for the preparation of related devices [28]. The average particle size is about 10 nm, and it is consistent with quantum size effect. In a certain range, the reaction activity of the PbS QD monomer was improved by the high temperature; consequently, the reaction was accelerated [29]. This helped to promote the formation of an excellent product.

Figure 2c exhibits the XRD result of the prepared product. The diffraction peaks of the product can be seen located at 26°, 30°, 43°, 51°, 53°, 62°, 69°, 71°, 79°, 84°, corresponding to crystal planes (111), (200), (220), (311), (222), (400), (331), (420), (422) and (511), respectively. This result is consistent with the standard PDF card (JCPDS Card No. 05-0592) and corresponds to the face-centered cubic structure of PbS QDs. The peak with the highest intensity shows that the grains are preferentially oriented along the (200) direction. From the result, all peaks are sharp and dominant, and there are no undesired peaks, which indicates that the product obtained in this research possesses good crystallinity [30]. Good crystallinity can reduce the internal structural defects of QDs and reduce the probability of electron recombination in the migration process between the crystal and QDs.

On account of the synthesis method in this research, the synthesized PbS QDs present a wide absorption range, the first absorption excitonic peak ranging from 1400 to 1600 nm (Figure 2d). This means that PbS QDs are able to absorb the light in this range, which will make the photodetector based on PbS QDs sensitive to the light in this range.

### 3.2. The Preparation of the Photodetector Based on PbS QDs and Graphene

**Figure 3:**
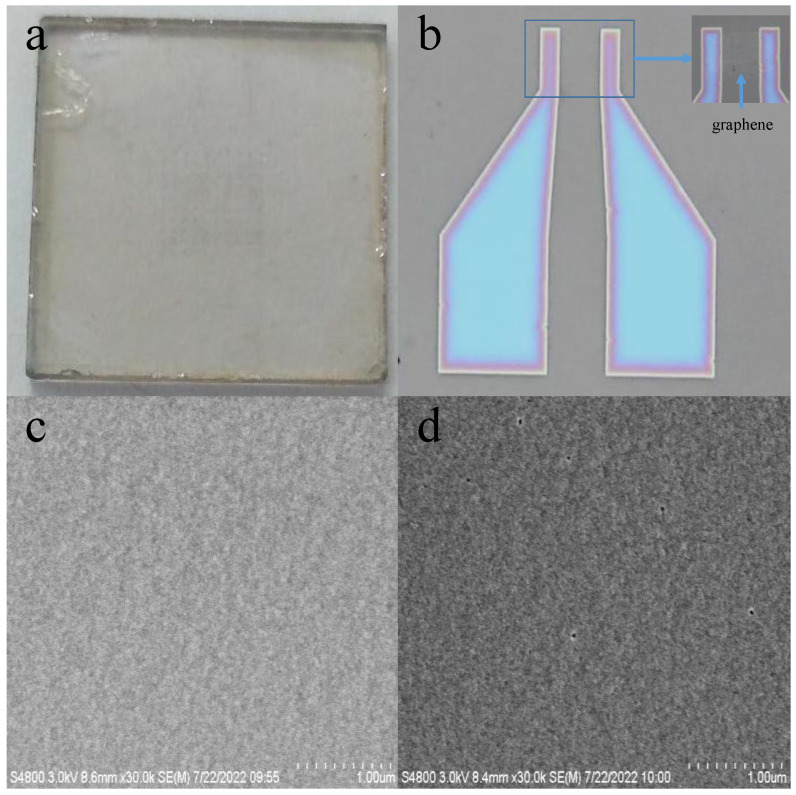
The assembly of the PbS QDs photodetector prepared in this research: (**a**) the appearance; (**b**) the micro morphology; (**c**) SEM image of the QD film on the device before substitution; (**d**) SEM image of the QD film on the device after substitution.

Figure 3a,b exhibits the assembly of the PbS QDs photodetector. ITO is a transparent brown or yellow block that is made of In_2_O_3_ and SnO_2_. Because it possesses outstanding electrical conductivity and light transmission properties, it is used widely in the field of photoelectric detection. For this reason, in this research, ITO was used as electrodes, which were obtained by photolithography and the HCl etching of ITO glass. The active region of the device was shaped by photolithography and the oxygen plasma etching of graphene. However, the responsivity of the device may be limited due to the weak light absorption of graphene [31], hence the combination of graphene and PbS QDs being actualized thanks to the strong photogating effect induced by trapped photocarriers in the QDs [32]. Therefore, the high photoconductive gain can be achieved by the high light absorption of PbS QDs and the high carrier mobility of graphene [33].

However, in the synthesis process of PbS QDs, a long-chain ligand is often introduced to control the nucleation and growth of the product [34]. A long-chain ligand can hinder the deposition of monomers on the surface of QDs to a certain extent, as it can adjust the size and regulate the shape of QDs by attaching to specific crystal surfaces. After its synthesis, the surface of the QDs is coated by long-chain ligands, and this can help the QDs to disperse stably in non-polar solvents and prevent them from aggregating with each other [35]. Because the surface ligand of QDs in devices affects various material parameters, including carrier density, mobility, chemical stability, charge transport, energy band gap structure, and so on. Therefore, the influence of ligands on the device can not be underestimated [36]. However, under the protection of a long-chain ligand, the interface barrier of QDs is too high, and the space between QDs is too large, hence the injection and transmission of carriers between QDs are hindered, which greatly reduces the performance of photoelectronic device [37,38]. Nevertheless, without additional modifications, the carrier’s mobility increases exponentially with the decrease in ligand length. This significant impact can be explained by the changes in dielectric environment and tunneling distance between QDs during ligand exchange. Therefore, it is necessary to replace the original long-chain ligand with a short-chain ligand to improve the carrier transport between QDs so as to improve the performance of the device [39,40]. It was for these reasons that in this research, OA was substituted for EDT to improve the conductivity of the device. It can be seem from Figure 3c,d that film formation occurred both before and after the substitution, and the good compactness and uniformity of the film improved the light absorption and carrier transmission capacity of the device.

### 3.3. The Performance of the Photodetector Based on PbS QDs and Graphene

Superior signal stability is also the key factor for the practical application of the photodetector. Therefore, the signal drift of the photodetector was also characterized in this research. Prior to the introduction of the incident light, the baseline current was 52.7 uA, and after the incident light was removed, the baseline current was 54.5 uA. Although there was a slight delay in the signal’s return to the previous baseline after the removal of the incident light, the device exhibited less signal drift (3.4%) and outstanding signal stability.

**Figure 4:**
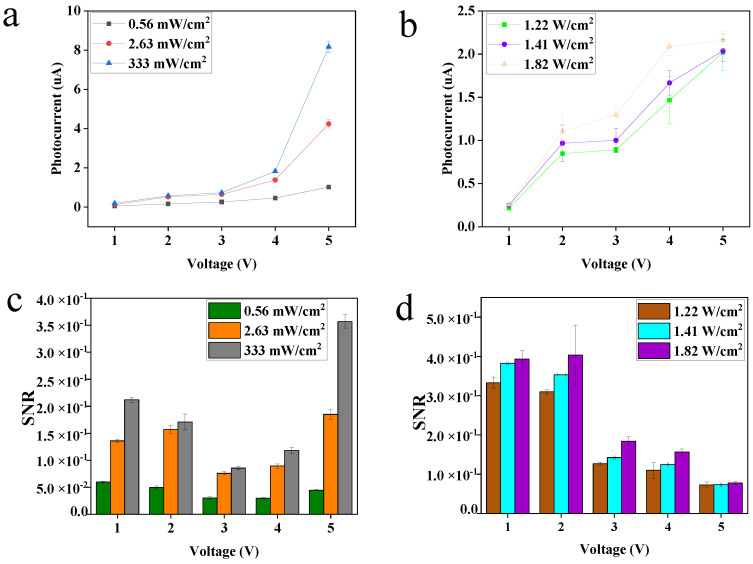
The photocurrent-voltage characteristics under (**a**) 405 nm and (**b**) 1550 nm. The signal-to-noise ratio under (**c**) 405 nmn and (**d**) 1550 nm.

The photocurrent–voltage (ΔI–V) characteristics of the PbS QD photodetector at different light wavelength with different light densities for an applied voltage from 1 V to 5 V are shown in Figure 4a,b. The photocurrent analysis at the same bias voltage shows that whether in visible or near-infrared light, the photocurrent increases with the increase in illumination. The light absorption, charge carrier generation, and transport led to increase in photocurrent [41]. Signal-to-noise ratio (SNR) is another parameter that can be used to measure the performance of the device, and it is defined as:

(1) $$SNR=\frac{I_{light-}I_{dark}}{I_{dark}}$$

where I_light_ is the light current, and I_dark_ is the dark light. SNR can reflect the information about the level of a desired signal from the background noise. The higher the value, the lower background noise [42]. The SNR results under visible and near-infrared light are shown in Figure 4c,d, and it can be seen that at the same voltage, under the condition of low light density, the SNR of the device is obviously lower than when under the condition of high light density. This phenomenon happened in both visible and near-infrared light. This can be explained by the fact that more carriers were generated as the light intensity increased, leading to a larger photocurrent, hence the larger SNR [43].

**Figure 5:**
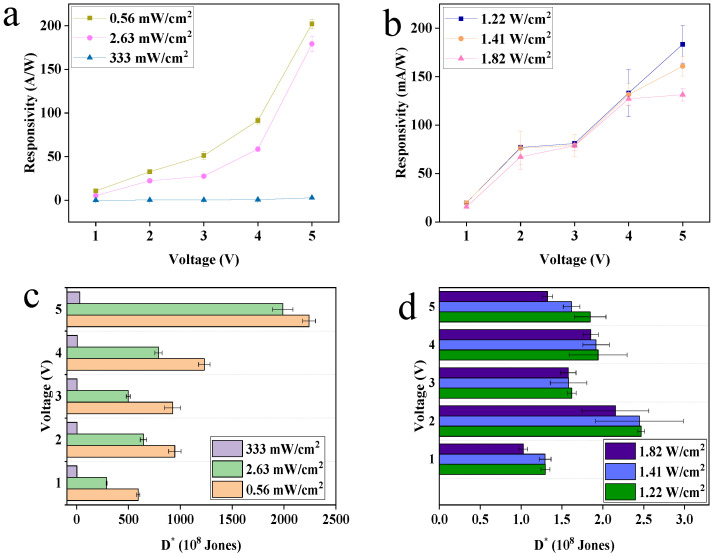
The responsivity under (**a**) 405 nm and (**b**) 1550 nm. The normalized detectivity under (**c**) 405 nmn and (**d**) 1550 nm.

For the photodetector, the detective responsivity R and normalized detectivity D* are other important parameters to determine whether the device can be applied in the reality. They are defined as

(2) $$R=\frac{I_{light}-I_{dark}}{P_{effective}}$$

(3) $$D^{*}=R\sqrt{\frac{S_{active}}{2qI_{dark}}}$$

where P_effective_ is the power of incident light on the active region, S_active_ is the area of the active region, and q is quantity of electric charge. From the results of visible light (Figure 5a) and near infrared light (Figure 5b), it is shown that the responsivity increases with the increase in voltage and decreases with the increase in light density, which is consistent with the research reported before [44]. As mentioned above, the higher the voltage, the larger the photocurrent Therefore, R increased with the increase in voltage due to an increase in photocurrent. However, the R value is negatively correlated with light intensity, and this is because the photoelectric conversion efficiency decreases with the increase in light intensity [45], meanwhile according to the formula, R is inversely proportional to light intensity. The R result is due to the above reasons, and the max R values of the two types of light are 202 A/W and 183 mA/W with light densities of 0.56 mW/cm^2^ and 1.22 W/cm^2^, respectively. In regard to D*, it can be seen that the D* results are both negatively correlated with light density not only in visible light but also in near-infrared light (Figure 5c,d), and at the same voltage, the max D* values occur when the light densities are lowest. This is attributed to the effect of deep level trap on the responsivity and detectivity. Because at a higher light intensity, the charge recombination loss is huge, hence the limited response and detection capabilities of the detector. 

**Figure 6:**
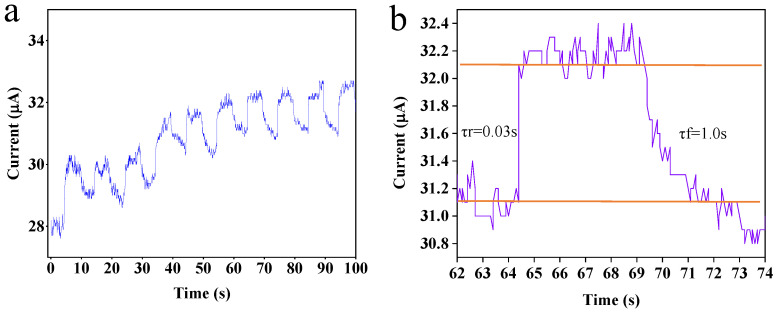
The response to on/off illumination cycles as well as the rise and fall times (τr/τf): (**a**) the whole time response data; (**b**) a single set of time response data.

In addition to the above parameters, the response of the photodetector to on/off illumination cycles is also vital to the examination of the application property of the device, and it was measured under a fixed bias of 1 V in this research. From the results (Figure 6), the dynamic responses and the tails in both the rise and fall are asymmetrical. This may be caused by the residual charge traps on the PbS QD surface and/or PbS QDs/graphene interface. Firstly, the photogenerated carriers were rapidly transferred to the electrode by the enhanced electric field under the bias and then slowly captured by the traps, thereby decaying to a stable value [46]. Under a chopper frequency of 0.1 Hz, the rise (10–90%) and fall (90–10%) times are about 0.03 s and 1.0 s, respectively. This faster response time of the photodetector based on graphene and PbS QDs is attributed to the photoinduced charge transfer between graphene and the PbS QDs [47].

Table 1 concluded the performance comparison of different photodetectors in the last few years. The results in the table shows that the performance of the photodetector from this work are better than other photodetectors, and shows a higher responsivity and detectivity.

## 4. Conclusions

In order to prepare the photodetector with excellent properties, in the early stage of this research, PbS QDs were synthesized and characterized by various methods to verify their excellent structures and properties. An ITO electrode and graphene active region were graphically processed by photolithography and etching to prepare the basic device. Later, a ligand substitution treatment was carried out after the QD product formed a film over the active region of the device. For the device, the electrode was prepared from ITO quartz substrate, which not only avoided the disadvantages of traditional silicon-based devices but also reduced the preparation cost of the device. Furthermore, the combination of graphene and PbS QDs was an excellent decision, as it has the advantages of the shortcomings of graphene making up for the superior photosensitive property and the wide spectral response to light of PbS QDs, and filling the deficiency of PbS QDs with the outstanding carrier mobility and quantum efficiency performances of graphene. This structure combined the respective advantages of the two materials to improve the performance of the device. Furthermore, the as-prepare graphene–PbS QDs photodetector was a combination of surface plasma structure and traditional photodetector, and this structure could generate a surface plasmonic effect under the excitation of corresponding incident light. Because of the plasmonic effect, the incident light was highly localized around the structure, thereby the interaction between light and absorbing matter was enhanced. Besides that, when the plasmonic effect was excited, the structure was in an enhanced local field, which induced the effective separation and collection of photocarrier. As a result, due to the improvement of carrier collection efficiency, the quantum efficiency of the device has been improved to a certain extent, and the detection efficiency of the photodetector has been improved.

Although the testing results verified that the photodetector, which was prepared in this research, showed both superior visible and near-infrared detection performance and stability, there were still some shortcomings that can still be overcome. For example, the introduction of a large number of defect states during the synthesis of PbS QDs is the key to affecting the performance of the device. Therefore, some modifications of parameters and methods can reduce the defect states of PbS QDs and upgrade the structure of the device. For instance, the optimization of ligand types, the design of energy level structures, and the improvement of the binding mode between PbS QDs and graphene. Improving the preparation and performance of these devices can be achieved by various methods, included those mentioned above.

## Figures and Tables

**Table 1:** The performance comparison of different photodetectors.

Wavelength (nm)	Responsivity (mA/W)	Detectivity (Jones)	References
UV–VIS–NIR	51	3.4 × 10^8^	[48]
1064	4.17	5.85 × 10^9^	[49]
980	2.3	3.31 × 10^10^	[50]
808	145	9.66 × 10^10^	[51]
2700	10.4 × 10^3^	2.98 × 10^9^	[52]
808	14 × 10^3^	3.9 × 10^8^	[53]
VIS–NIR	202 × 10^3^	2.24 × 10^11^	This research

## Data Availability

The data that support the findings of this study are available from the corresponding author upon reasonable request.
